# Sleep-disordered breathing is a risk factor for delirium after cardiac surgery: a prospective cohort study

**DOI:** 10.1186/s13054-014-0477-1

**Published:** 2014-09-05

**Authors:** Jens Roggenbach, Marvin Klamann, Rebecca von Haken, Thomas Bruckner, Matthias Karck, Stefan Hofer

**Affiliations:** Department of Anaesthesiology and Intensive Care Medicine, University of Heidelberg, University Hospital, Im Neuenheimer Feld 110, 69120 Heidelberg, Germany; Department of Medical Biometry and Informatics, University of Heidelberg, Im Neuenheimer Feld 305, 69120 Heidelberg, Germany; Heart Centre Heidelberg, Clinic for Cardiac Surgery, University of Heidelberg, University Hospital, Im Neuenheimer Feld 110, 69120 Heidelberg, Germany

## Abstract

**Introduction:**

Delirium is a frequent complication after cardiac surgery. Although various risk factors for postoperative delirium have been identified, the relationship between nocturnal breathing disorders and delirium has not yet been elucidated. This study evaluated the relationship between sleep-disordered breathing (SDB) and postoperative delirium in cardiac surgery patients without a previous diagnosis of obstructive sleep apnea.

**Methods:**

In this prospective cohort study, 92 patients undergoing elective cardiac surgery with extracorporeal circulation were evaluated for both SDB and postoperative delirium. Polygraphic recordings were used to calculate the apnea-hypopnea index (AHI; mean number of apneas and hypopneas per hour recorded) of all patients preoperatively. Delirium was assessed during the first four postoperative days using the Confusion Assessment Method. Clinical differences between individuals with and without postoperative delirium were determined with univariate analysis. The relationship between postoperative delirium and those covariates that were associated with delirium in univariate analysis was determined by a multivariate logistic regression model.

**Results:**

The median overall preoperative AHI was 18.3 (interquartile range, 8.7 to 32.8). Delirium was diagnosed in 44 patients. The median AHI differed significantly between patients with and without postoperative delirium (28 versus 13; *P* = 0.001). A preoperative AHI of 19 or higher was associated with an almost sixfold increased risk of postoperative delirium (odds ratio, 6.4; 95% confidence interval, 2.6 to 15.4; *P* <0.001). Multivariate logistic regression analysis showed that preoperative AHI, age, smoking, and blood transfusion were independently associated with postoperative delirium.

**Conclusions:**

Preoperative SDB (for example, undiagnosed obstructive sleep apnea) were strongly associated with postoperative delirium, and may be a risk factor for postoperative delirium.

**Electronic supplementary material:**

The online version of this article (doi:10.1186/s13054-014-0477-1) contains supplementary material, which is available to authorized users.

## Introduction

Postoperative delirium is a frequent complication after cardiac surgery [1,2]. Delirium is a major risk factor for an increased length of stay in the intensive care unit, and is associated with an increased mortality rate and increased costs [3,4]. The incidence of delirium after cardiac surgery is reported to be between 3% and 72% overall [5], and between 30% and 50% in older patients [1,4,6,7]. Numerous studies reported that age, functional status, preoperative cognitive impairment, depression, smoking, alcohol abuse, drug abuse, history of stroke, diabetes, hematological changes (for example, anemia), low albumin level, intraoperative blood transfusion, and atrial fibrillation were associated with postoperative delirium [1,5,6,8-11]. Although obstructive sleep apnea syndrome (OSAS) appears to be associated with cardiovascular disease, stroke, and cognitive impairment (for example, deficits in memory, attention, vigilance, and learning) [12,13], sleep-disordered breathing (SDB) has only infrequently been considered as a potential risk factor for postoperative delirium [14,15]. The most prevalent form of SDB is OSAS, which is characterized by intermittent loss of airway patency during sleep with sleep fragmentation, increased nocturnal endogenous stress, intermittent oxygen desaturations and the loss of a normal sleep architecture. OSAS induces inflammatory pathways and is associated with endothelial dysfunction, probably due to diminished nitric oxide synthesis, which could result in vascular damage and could therefore contribute to cardiovascular disease and vascular encephalopathy [16]. It therefore appears likely that OSAS is a risk factor for postoperative cognitive impairment. It was recently reported that a history of OSAS was associated with postoperative delirium after knee replacement surgery [17]. It is noteworthy that OSAS is undiagnosed in the majority of patients, and it is therefore likely that undiagnosed SDB is highly prevalent in the older population and in individuals with cardiovascular disease [12,18-20]. The aim of this study was to investigate the relationship between SDB and postoperative delirium in patients undergoing cardiac surgery who did not have a previous diagnosis of OSAS.

## Materials and methods

### Subjects

The Institutional Ethics Committee of the University of Heidelberg approved the study protocol. The recruitment period was from September 2011 until January 2012. Patients undergoing elective cardiac surgery were screened for eligibility, and informed consent was obtained from eligible patients who were willing to participate in the study. The inclusion criteria were: age >18 years, no previous diagnosis of sleep apnea, no history of cognitive impairment, and undergoing elective coronary artery surgery or heart valve replacement/repair either with or without coronary bypass grafting. Of the 130 patients who were asked to participate in this study, 16 declined and 114 agreed. Twenty-two patients were excluded due to insufficient polygraphic recordings. The final study sample consisted of the remaining 92 patients.

The medical history of each patient was obtained from their medical chart. Smoking was defined as any current smoking or a history of smoking with a cessation of less than six months. Alcohol abuse was defined as an average alcoholic intake of two or more drinks or equivalent daily. Laboratory data collected included hematological data and blood gas analysis. Intraoperative data such as operation time, extracorporal circulation time, cross-clamp time, transfusions, and administered drugs were collected from the anesthesia protocol and the perfusionists’ records.

As a standard dose for premedication, all patients received oral midazolam 7.5 mg as premedication, reduced to 3.75 mg if they were aged >70 years or their body mass was <50 kg. After placement of an arterial line, anesthesia was induced with a standardized regimen of sufentanil, etomidate, and pancuronium. In patients with a suspected risk of aspiration or renal failure, rocuronium and cisatracurium, respectively, was used for relaxation. Anesthesia was maintained with sufentanil and sevoflurane pre and post bypass, and with propofol during extracorporal circulation. Details of the maintenance of anesthesia were at the discretion of the anesthesiologist. After surgery, all patients were transferred to the intensive care unit while intubated and sedated. Postoperative sedation was standardized, using clonidine and opioids (usually piritramide, with an analgesic potency relative to morphine of 0.7). Extubation time was at the discretion of the intensive care physician. By default, postoperative therapy with continuous positive airway pressure (CPAP) was applied in patients with postoperative atelectasis, pleural effusion and after a prolonged intubation time (>48 hrs.). CPAP therapy was usually applied with a set pressure of 5 mbar and a pressure support of 10 mbar, adjusted to an inspiratory tidal volume of 6 to 8 ml/kg body weight. Overnight CPAP was not applied during the postoperative period.

### Polygraphic recordings

Nocturnal polygraphic recordings of breathing patterns were obtained using portable polygraphs (Mini-Screen 4, Heinen and Löwenstein, Bad Ems, Germany). The Mini-Screen 4 polygraph used pulse oximetry to measure heart rate and oxygen saturation, and a nasal pressure probe to measure respiration and snoring. Measurements were obtained during the night before surgery. Data regarding relevant coexisting diseases, long-term medications, and administered analgesics and sedatives were collected for each patient. The polygraphic recordings were evaluated by a trained physician, who was unaware of the history and perioperative course of the patients. Sleep-associated respiratory events were classified based on the recommendations for measurement techniques in clinical research of the American Academy of Sleep Medicine [21]. An apnea was scored when respiratory flow dropped by ≥90% of baseline for at least 10 seconds, and a hypopnea was scored when nasal pressure signal excursions dropped by ≥50% for at least 10 seconds accompanied by an oxygen desaturation of at least 3%. The apnea-hypopnea index (AHI) was calculated as the mean number of apneas and hypopneas per hour recorded. Episodes with artifacts during polygraphic recordings, restricting reliable evaluation were deleted. Exclusion criteria for nocturnal measurements were non-evaluable polygraphic recordings or a recording time of less than four hours.

### Assessment of delirium

Delirium was diagnosed using the Confusion Assessment Method for the intensive care unit (CAM-ICU) [22]. The CAM-ICU is a four-item, easily applicable delirium screening instrument, which can be performed by trained non-clinicians. A positive diagnosis requires as a first criterion an acute alteration or a fluctuating course of the mental state. If the first criterion is given, patients are checked for attention deficits as a second criterion. The CAM-ICU is considered positive if criterion 1 and 2 are given in combination with either disorganized thinking or a change in consciousness (see Additional file 1). Before assessment of the CAM-ICU, the sedative state was evaluated as a standard feature using the Richmond Agitation and Sedation Scale (RASS) [23,24]. In patients with deep sedative stages, rated as a RASS-score of −4 or −5, evaluation was omitted. All participants were examined twice a day when RASS score was ≥ −3 for the following four postoperative days either by a trained researcher or an intensive care physician. A CAM-ICU worksheet shows this more in detail (see Additional file 1).

### Statistical analysis

Statistical analyses were performed using SPSS software, version 19.0 (IBM Corp, Armonk, NY, USA). The normality of data distribution was determined using the Kolmogorov-Smirnov test. Patient characteristics are expressed as mean and standard deviation for normally distributed data and as median, interquartile range (IQR) and range for non-normally distributed data. Comparisons between groups were performed using the Mann-Whitney *U* test or Student’s *t* test, as appropriate. The significance of differences between groups was tested using the Pearson’s chi-square test for categorical data. A multivariate logistic regression analysis was performed to determine the relationship between postoperative delirium, SDB and those covariates that were found to be associated with delirium in previous studies. As a prerequisite for entering the logistic regression model, all covariates had to differ significantly in individuals with delirium as compared to individuals without postoperative delirium in univariate analysis. The AHI value with the highest accuracy of discrimination between individuals with and without postoperative delirium was determined with a receiver operating characteristic (ROC) curve. A value of *P* = 0.05 was considered significant in all analyses.

## Results

Characteristics of the study sample are presented in Table 1 and results of the polygraphic recordings are shown in Table 2. Postoperative delirium was diagnosed in 44 of the 92 patients, included in this study. Delirium occurred most commonly on the first and second postoperative day, and lasted for a median of 1.3 days (range 1 to 4 days). After extubation all patients received piritramide for analgesia and clonidine for sedation as needed by default. Before a diagnosis of delirium was made patients with delirium did not differ in their use of analgesics and sedatives as compared with patients who did not develop delirium postoperatively. Patients with postoperative delirium, spent significantly more time in the intensive care unit (median 21 versus 46 hrs; *P* <0.001) and intermediate care unit (median 6 days versus 4 days; *P* <0.001) than patients without postoperative delirium.

**Table 1:** **Patient characteristics, according to postoperative diagnosis of delirium**

	**All patients (n = 92)**	**No postoperative delirium (n = 48)**	**Postoperative delirium (n = 44)**	***P***
Male, n (%)	66 (60.6)	35 (72.9)	31 (70.4)	0.082
Body mass index (BMI)	27.1 ± 3.9	27.1 ± 3.3	27.2 ± 4.5	0.91
Mean age, (years)	67.5 ± 8.9	64.5 ± 9	70.8 ± 7.8	0.001
Alcohol abuse, n (%)	10 (10.9)	3 (6.2)	7 (15.9)	0.14
Smoking, n (%)	23 (25)	7 (14.6)	16 (36.3)	0.016
Diabetes, n (%)	21 (22.8)	10 (20.8)	11 (25)	0.63
Arterial hypertension, n (%)	89 (96.7)	46 (95.8)	43 (97.7)	0.34
Chronic obstructive lung disease, n (%)	20 (21.7)	7 (14.6)	13 (29.5)	0.09
Renal insufficiency, n (%)	13 (14.1)	5 (10.4)	8 (18.2)	0.25
Impaired left ventricular function, EF n (%): normal (≥55%)	58 (63)	30 (62.5)	28 (65.1)	0.7
Mild (45-54%)	16 (17.4)	10 (20.8)	5 (11.6)	
Moderate (36-44%)	8 (8.7)	3 (6.3)	4 (9.3)	
Severe (≤35%)	11 (12.5)	5 (10.4)	6 (14)	
History of stroke, n (%)	7 (7.6)	3 (6.3)	4 (9.1)	0.48
Atrial fibrillation preoperative, n (%)	13 (14.1)	6 (12.5)	7 (16.7)	0.22
Long-term glucocorticoid medication, n (%)	5 (5.4)	0 (0.0)	5 (11.4)	0.056
Albumin g/l	43.5 ± 3.4	44.4 ± 3.1	42.3 ± 3.4	0.003
Hemoglobin g/dl	13.2 ± 1.6	13.7 ± 1.5	12.7 ± 1.6	0.005
Urea mg/dl	37.9 ± 19.0	36.1 ± 13.9	40.2 ± 23.5	0.33
Mean number of transfused packs of RBC	3.0 ± 2.3	2.2 ± 1.8	3.8 ± 2.6	0.001
Type of heart surgery (CABG/open heart surgery ± CABG)	47/45	26/22	21/23	0.54
Mean extracorporeal circulation time (min.)	112 ± 41.5	108 ± 41.4	115 ± 42	0.37
Postoperative intubation time (hrs) median (IQR [range])	13 (10-18 [4-91])	11 (9-14 [5–29])	18 (14-23[4-91])	<0.001
Time (days) spent in intensive and intermediate care unit, median (IQR [range])	5 (3-6 [2–17])	4 (3-5 [2–15])	6 (5-8 [2–17])	<0.001
Time (hrs) spent in intensive care unit, median (IQR [range])	23 (20-47 [11-289])	21 (19-24 [12-77])	46 (23-78[11-289])	<0.001
Benzodiazepine intake during the night of the sleep study (yes/no)	22 (25.6)	12 (27.9)	10 (25.6)	0.82

(EF, ejection fraction; RBC, red blood cell; CABG, coronary artery bypass grafting; IQR, interquartile range; data in cells represent absolute values or mean ± standard deviation).

**Table 2:** **Preoperative nocturnal breathing characteristics of examined patients**

	**All patients (n = 92)**	**No postoperative delirium (n = 48)**	**Postoperative delirium (n = 44)**	***P***
AHI; median (IQR [range])	18.3 (8.7-32.8 [2-65])	13.2 (7.1-22.4 [2–51])	27.7 (14.0-39.7 [2-65])	0.001
AHI, n (%):				<0.01
<5	9 (9.8)	6 (12.5)	3 (6.8)	
≥5	83 (90.2)	42 (87.5)	41 (93.2)	
≥15	54 (58.7)	21 (43.8)	33 (75)	
≥30	29 (31.5)	8 (16.7)	21 (47.7)	
Mean oxygen saturation; mean ± SD	92.3 ± 1.8	92.5 ± 1.9	92.1 ± 1.6	0.36
Mean oxygen desaturation; mean ± SD	91.7 ± 1.9	92.2 ± 1.8	91.0 ± 1.8	<0.01
Mean minimal oxygen saturation; mean ± SD	77.9 ± 6.3	79.1 ± 5.9	76.3 ± 6.6	0.07
Measured time (in%) spent with an oxygen saturation <90%; mean ± SD	5.8 ± 11.8	4.1 ± 11.7	7.8 ± 11.8	0.17

(Mean oxygen saturation describes the overall mean oxygen saturation during the night. Mean oxygen desaturation is defined as the mean decrease in oxygen saturation during apnea/hypopnea. Mean minimal oxygen saturation describes the mean values of the lowest oxygen saturation measured during the recording time. IQR, interquartile range, data in cells mean ± SD, standard deviation). AHI, apnea-hypopnea index.

The median AHI of the study population was 18.3 during the index night and differed significantly between patients with and without postoperative delirium (Table 2). Ninety percent of all patients had a preoperative AHI of at least 5, 59% had an AHI of 15 or higher and an AHI of at least 30 was found in almost 32% of all patients. Comparing patients with and without postoperative delirium, showed that a preoperative AHI of least 15 and 30, respectively, was substantially more prevalent in the group of patients with postoperative delirium. An AHI of at least 15 was found in 75% and 44% of all patients with and without delirium. Forty-eight percent of all patients with postoperative delirium had an AHI of at least 30, while an AHI of 30 or higher was found in 17% of all patients without postoperative delirium (*P* <0.01, Table 2).

**Figure 1:**
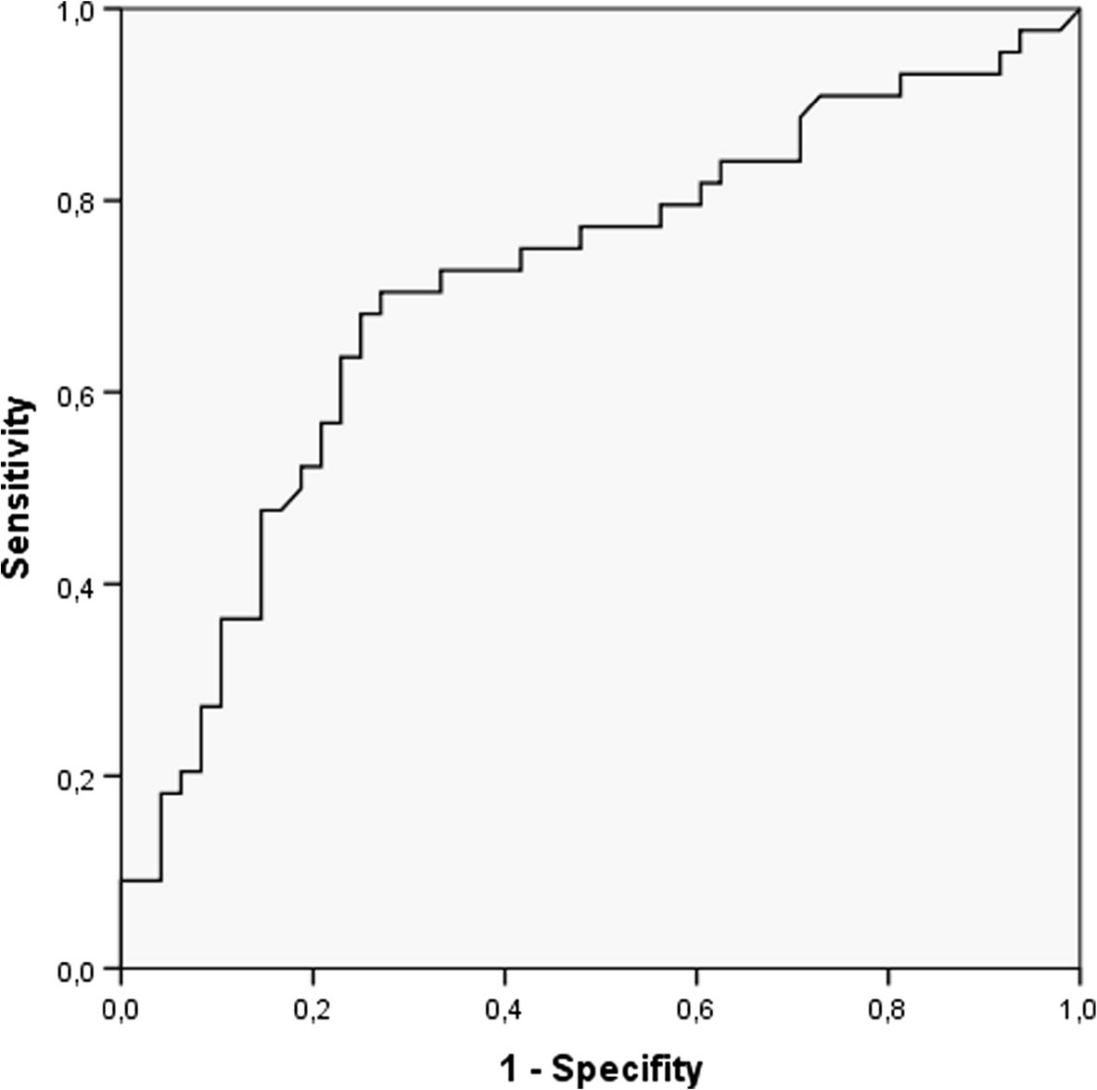
**Receiver operating characteristic (ROC) curve of the apnea-hypopnea index of predictability for postoperative delirium.** The area under the curve (AUC) was 0.712 (confidence interval: 0.604 to 0.82) with a cutoff value of 19 (sensitivity 71%, specificity 73%).

**Figure 2:**
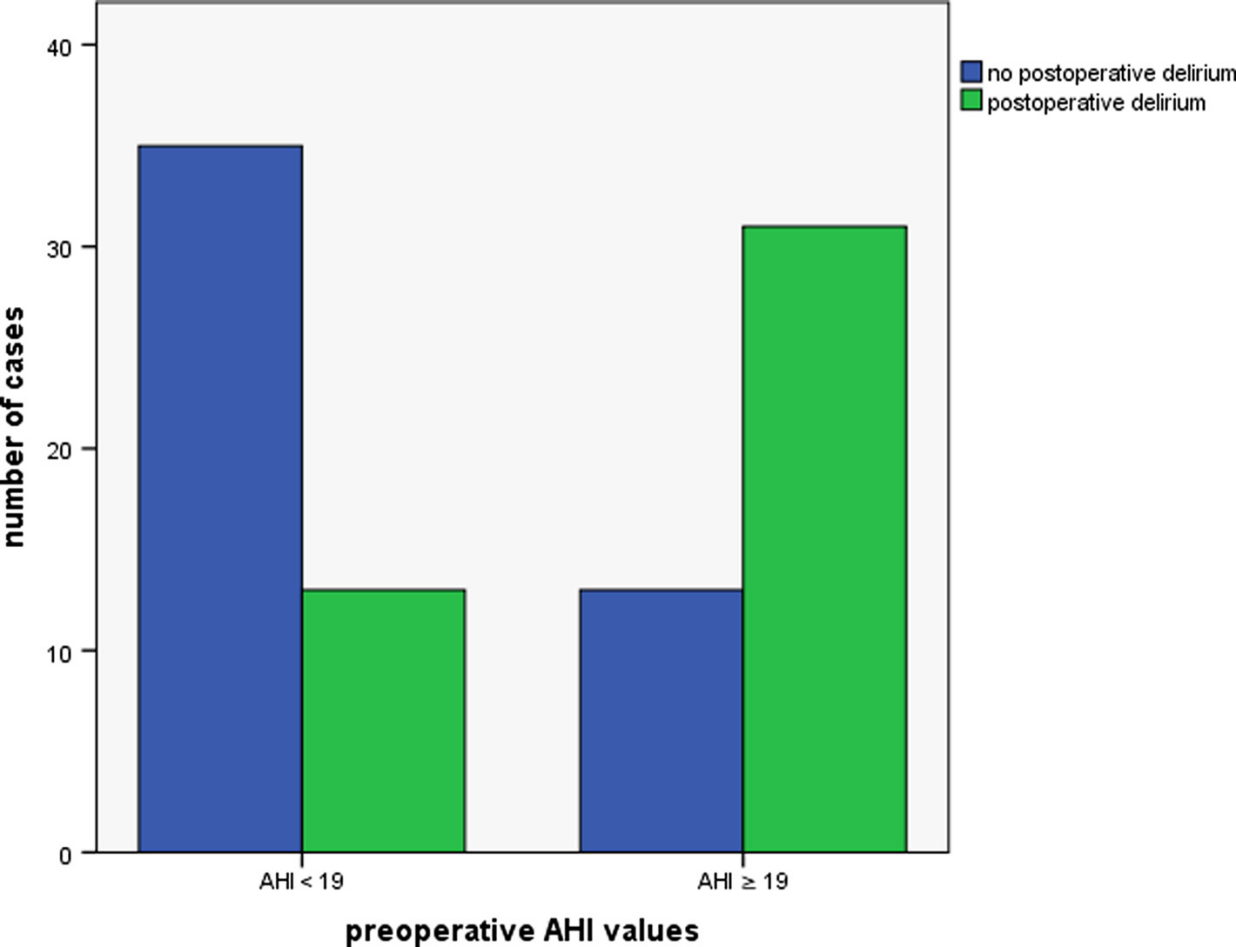
**Bar chart indicating the number of cases of individuals with postoperative delirium, divided into groups with preoperative apnea-hypopnea index (AHI) values higher or lower than 19.**

The AHI value with the highest accuracy for discrimination between individuals that did or did not develop postoperative delirium was derived from a ROC curve (Figure 1). A preoperative AHI of 19 or higher was associated with a sixfold increased risk of postoperative delirium (odds ratio, 6.4; 95% confidence interval, 2.6 to 15.4; *P* <0.001; Figure 2). Patients with postoperative delirium had a more pronounced mean oxygen desaturation during apnea/hypopnea as compared with patients without delirium. The mean time that individuals spent with an oxygen saturation below 90% during the index episode, however, showed no statistical difference between the groups (Table 2).

Patients with postoperative delirium were significantly older, had lower preoperative hemoglobin levels and lower preoperative serum albumin levels than patients without postoperative delirium. Patients with postoperative delirium also received significantly more units of blood transfusion during surgery than patients without postoperative delirium. A history of smoking was more frequent in individuals who developed postoperative delirium, but there was no significant association with alcohol abuse. Chronic glucocorticoid intake was more prevalent in patients with postoperative delirium than those without postoperative delirium.

Precise data regarding sedative intake during the night before surgery was available for only 82 patients, of whom 22 received benzodiazepines. Preoperative benzodiazepine use was not associated with postoperative delirium. The median AHI was not significantly different between patients who received benzodiazepines and those who did not (25.5 (11.9 to 36.4 [2 to 65]) versus 18.3 (9.7 to 34.7 [2 to 57]; *P* = 0.33).

Multivariate logistic regression analysis including preoperative AHI, age, preoperative albumin and hemoglobin values, and intraoperative blood transfusion as continuous variables and smoking as a categorical variable showed that, after adjustment for the covariates, preoperative AHI, age, smoking and intraoperative blood transfusions were significantly associated with postoperative delirium, with a degree of classification of 77.8% (Table 3).

**Table 3:** **Logistic regression for postoperative delirium as a dependent variable**

	**Regression coefficient**	**Wald**	***P***	**Exp (B)**	**95% CI for Exp (B)**
AHI	0.05	5.45	0.02	1.05	1.01-1.1
Smoking	3.48	10.40	0.001	32.37	3.9-268
Age	0.15	6.53	0.011	1.16	1.04-1.29
Intraoperative blood transfusion	0.44	4.43	0.04	1.55	1.03-2.32
Preoperative hemoglobin	0.24	0,81	0.37	1.27	0.75-2.16
Albumin	−0.12	1.29	0.26	0.89	0.72-1.09

(Exp (B) describes the change in odds ratio for each unit change of the independent variable). AHI, apnea-hypopnea index; CI, confidence interval.

During the postoperative course five patients required re-operation due to pericardial effusion. One patient needed postoperative treatment with an intra-aortic balloon pump. Two patients had to be resuscitated due to cardiac failure and two patients died (one with sepsis and one with multiple organ failure). Intermittent CPAP therapy was very common in the postoperative period, mainly in patients with postoperative atelectasis, pleural effusion and following a prolonged intubation time. Intermittent postoperative CPAP therapy was applied to 36 patients. Patients with postoperative delirium were treated significantly more often with CPAP therapy as compared to individuals without postoperative delirium (57% vs. 25%, *P* <0.01). Severity of preoperative AHI values was not associated with postoperative CPAP therapy.

## Discussion

In this prospective study we found that a preoperative AHI of 19 or higher was associated with a sixfold increased risk of postoperative delirium. After adjustment for relevant covariates, multivariate logistic regression analysis revealed that AHI, age, smoking, and blood transfusion remained significant independent predictors of postoperative delirium.

Numerous studies have reported various risk factors for postoperative delirium after cardiac surgery [1]. Among these, age, depression, history of stroke, cognitive impairment, diabetes mellitus and atrial fibrillation have been frequently identified as potential risk factors for postoperative delirium [1]. Our findings that age, smoking and intraoperative blood transfusion was associated with postoperative delirium are in accordance with the findings of other studies [1,7,25-27]. Nicotine abuse is, similar to advanced age, associated with an increased risk for atherosclerosis, which has been found to be an independent risk factor for postoperative delirium after cardiac surgery [28]. The prevalence of diabetes, atrial fibrillation and history of stroke was probably too low in this study cohort to confirm the observations of other studies.

SDB has only infrequently been considered as a potential risk factor for postoperative delirium. A recent study found that patients with a previous diagnosis of obstructive sleep apnea were more likely to develop postoperative delirium after knee replacement surgery than those without a diagnosis of sleep apnea [17]. Remarkably, SDB and OSAS are very common among adults and their prevalence increases with age [29]. Previous studies have reported an association between OSAS and heart failure [30], and a high prevalence of SDB among patients with acute coronary syndrome [31,32]. However, older patients are less likely to experience symptoms related to obstructive sleep apnea (for example, daytime sleepiness, snoring), and it has to be assumed that the vast majority of sleep-associated breathing disorders are undiagnosed in this group of patients [19,29,32-34].

SDB events were very common in our study group. As expected, preoperative AHI was correlated with age and body mass index (BMI). Our findings are consistent with those of epidemiological studies showing a high prevalence of sleep-related breathing disorders and OSAS in individuals >65 years [19,34] and in individuals with coronary artery disease [32,35,36].

Various studies have reported an association between obstructive sleep apnea and cognitive impairments such as deficits in attention, vigilance, memory, and learning [12,13], which probably increase the susceptibility of patients with OSAS to postoperative deterioration in mental state. The pathophysiology of cognitive impairment in patients with OSAS is still not well understood, but it is probably multifactorial. It has been suggested that the cognitive decline in patients with OSAS is associated with oxidative stress and reperfusion injury due to repetitive hypoxemia [13]. In this study, we did not find significant differences in mean nocturnal oxygen saturation or the mean proportion of time with oxygen saturation <90% between patients with and without postoperative delirium. Patients with postoperative delirium had a more pronounced decline in mean oxygen saturation during apnea and hypopnea than patients without delirium, but this difference was small (91% versus 92.2%), and the clinical relevance of this finding is therefore unclear.

Chronic OSAS results in endothelial dysfunction with a preponderance of vasoconstrictive mediators, reduced nitric oxide bioavailability, inflammatory stress, hypercoagulability, and increased risk of atherosclerosis [13,18], which could result in stroke and vascular dementia. Interestingly, inflammatory mediators (for example, interleukin-1, interleukin-6, and tumor necrosis factor alpha (TNF-α)) can contribute to delirium [37,38]. As cardiac surgery with extracorporeal circulation induces a systemic inflammatory reaction [39,40], patients with sleep-associated respiratory disorders may be particularly susceptible to neurological injury following cardiopulmonary bypass.

An association between OSAS, encephalopathy and postoperative delirium has been previously described in cardiac and noncardiac surgical patients [17,41]. In a study by Flink *et al*. [17] of 106 patients undergoing elective knee replacement, a history of OSAS was the only predictor of postoperative delirium. As pointed out by the authors, since OSAS is vastly undiagnosed, the association of OSAS and postoperative delirium might be even more pronounced than shown by their data.

In another study, Kaw *et al*. [41] identified 37 individuals with a history of OSAS from a database of cardiac surgical patients. These patients were matched with 185 cardiac surgical patients without a diagnosis of OSAS. The authors found a higher incidence of encephalopathy and a higher infection rate in patients with a history of OSAS. Unfortunately, encephalopathy was not further specified and it is unclear how many of these patients were delirious. Additionally, as discussed by the authors, the prevalence of OSAS in the control group was unknown.

When investigating the impact of OSAS on perioperative outcome, it has to considered that patients with a diagnosis of OSAS frequently use their home CPAP, which in turn has the potency to improve cognitive impairment, relieve depressive symptoms and attenuates the chronic inflammatory process [13,42-45]. Furthermore, compliant use of CPAP reduces mortality and hospitalization rate in patients with OSAS [18,46]. Consequently, the perioperative complication rate might be even lower in the small subgroup of patients with a previous diagnosis of OSAS compared with the majority of patients having undiagnosed OSAS. This, however, might contribute to the underestimation of OSAS-associated perioperative complications when comparing individuals with a previous diagnosis of OSAS with patients without such a diagnosis. In contrast to previous studies, to avoid the bias of preoperative home CPAP usage, we have chosen to include only individuals without a previous diagnosis of OSAS in this study.

The prevalence of undiagnosed OSAS is extremely high, especially in individuals with cardiac diseases, and thus, we felt that the investigation of SDB in cardiac surgical patients without a previous diagnosis of OSAS is of utmost importance. The preoperative polygraphic evaluation of all patients allowed us to evaluate the relationship between the severity of SDB and postoperative delirium. Additionally, it considers the high prevalence of undiagnosed SDB and thus, avoids the potential confounder of an unknown prevalence of OSAS and SDB in a control group with which studies are confronted, comparing patients with and without a previous diagnosis of OSAS.

A limitation of this study is the use of portable polygraphs, instead of polysomnography to evaluate sleep-disordered breathing. The polygraphs used in this study did not provide a channel to measure thoracic wall motion, and thus an unequivocal differentiation between central and obstructive apnea/hypopnea was not possible. Furthermore, the polygraph used in this study cannot determine total sleep time. Thus, AHI was calculated with recording time, which might underestimate AHI severity, especially in the presence of high AHI values [47]. While in various studies a high diagnostic accuracy for detecting SDB and a good correlation of AHI values has been shown for polygraphs compared with polysomnography [47-52], it is conceivable that the patients in this study population did not sleep well during the night before surgery and thus, the polygraphs might have underestimated the AHI scores in this setting.

Another limitation of this study is the missing assessment of preoperative cognitive function, since even milder degrees of cognitive impairment have been described as a predisposing risk factor for postoperative delirium [4,7,10]. The association OSAS with cognitive dysfunction has been described previously [12,13] and the examination of the relationship of SDB, preexisting cognitive impairment and delirium following cardiac surgery warrants further studies.

## Conclusions

This study identified nocturnal breathing disorders, age, smoking, and intraoperative blood transfusion as independent risk factors for postoperative delirium after cardiac surgery. Sleep-associated breathing disorders have not been well studied as a potential risk factor for delirium, and the results of this study highlight an important area for delirium research. As sleep-associated breathing disorders are highly prevalent among older people, screening for SDB might be useful to identify individuals at risk for postoperative delirium. It is tempting to speculate that continuous positive airway pressure therapy for these patients might prevent and treat postoperative delirium. Further investigations are necessary to elucidate the emerging questions in this field of delirium research.

## Key messages

- Sleep-disordered breathing is highly prevalent in individuals undergoing cardiac surgical procedures.
- An apnea-hypopnea index of 19 or higher is associated with a sixfold increased risk of postoperative delirium.

## Additional file

Additional file 1:
**CAM-ICU worksheet. The CAM-ICU worksheet is a concise description on how to perform the CAM-ICU in a standardized manner.**

## Abbreviations

AHI: apnea-hypopnea index: total number of apneas and hypopneas per hour sleep.
CAM-ICU: confusion assessment method for the intensive care unit: delirium monitoring instrument
CPAP: continuous positive airway pressure
IQR: interquartile range
OSAS: obstructive sleep apnea syndrome: defined as an AHI >5 combined with associated symptoms (for example fatigue, day-time sleepiness) or an AHI >15, irrespective of any symptoms
RASS: Richmond Agitation and Sedation Scale: sedation monitoring instrument
ROC: receiver operating characteristic
SDB: sleep-disordered breathing: sleep-associated abnormal breathing patterns (pauses in breathing or reduced ventilation during sleep)
