# Supplementary material for: Sleep-disordered breathing is a risk factor for delirium after cardiac surgery: a prospective cohort study
Source: Crit Care. 2014 Sep 5;18(5):477. doi: 10.1186/s13054-014-0477-1 (PMC4175616; doi:10.1186/s13054-014-0477-1)

Appendix 1

CAM-ICU Worksheet

CAM-ICU Worksheet

**Feature 1: Acute Onset or Fluctuating Course**<br>Positive if you answer 'yes' to either 1A or 1B.	**Positive**	**Negative**
**1A:** Is the pt different than his/her baseline mental status?<br>Or<br>**1B:** Has the patient had any fluctuation in mental status in the past 24 hours as evidenced by fluctuation on a sedation scale (e.g. RASS), GCS, or previous delirium assessment?	**Yes**	**No**
**Feature 2: Inattention**<br>Positive if either score for 2A <u>or</u> 2B is less than 8.<br>Attempt the ASE letters first. If pt is able to perform this test and the score is clear, record this score and move to Feature 3. If pt is unable to perform this test <u>or</u> the score is unclear, then perform the ASE Pictures. If you perform both tests, use the ASE Pictures' results to score the Feature.	**Positive**	**Negative**
**2A: ASE Letters:** record score (enter NT for not tested)<br><br><u>Directions</u>: Say to the patient, *"I am going to read you a series of 10 letters. Whenever you hear the letter 'A,' indicate by squeezing my hand."* Read letters from the following letter list in a normal tone.<br>**S A V E A H A A R T**<br>Scoring: Errors are counted when patient fails to squeeze on the letter "A" and when the patient squeezes on any letter other than "A."	**Score (out of 10): ______**	
**2B: ASE Pictures:** record score (enter NT for not tested)<br>Directions are included on the picture packets.	**Score (out of 10): ______**	
**Feature 3:Disorganized Thinking**<br>Positive if the combined score is less than 4	**Positive**	**Negative**
**<u>3A: Yes/No Questions</u>**<br>(Use either Set A <u>or</u> Set B, alternate on consecutive days if necessary)**:**<br>**Set A**<br>1. Will a stone float on water?<br>2. Are there fish in the sea?<br>3. Does one pound weigh more than two pounds?<br>4. Can you use a hammer to pound a nail?<br>**Set B**<br>1. Will a leaf float on water?<br>2. Are there elephants in the sea?<br>3. Do two pounds weigh more than one pound?<br>4. Can you use a hammer to cut wood?<br><br>**Score** ___(Patient earns 1 point for each correct answer out of 4)<br><br>**<u>3B:Command</u>**<br>Say to patient: "Hold up this many fingers" (Examiner holds two fingers in front of patient) "Now do the same thing with the other hand" (Not repeating the number of fingers). *If pt is unable to move both arms, for the second part of the command ask patient "Add one more finger)<br><br>**Score**___(Patient earns 1 point if able to successfully complete the entire command)	**Combined Score (3A+3B):**<br>**_____ (out of 5)**	
**Feature 4: Altered Level of Consciousness**<br>Positive if the Actual RASS score is anything other than "0" (zero)	**Positive**	**Negative**
**Overall CAM-ICU** (Features 1 and 2 and either Feature 3 or 4):	**Positive**	**Negative**

Copyright © 2002, E. Wesley Ely, MD, MPH and Vanderbilt University, all rights reserved

Visual ASE - Packet A

Step 1

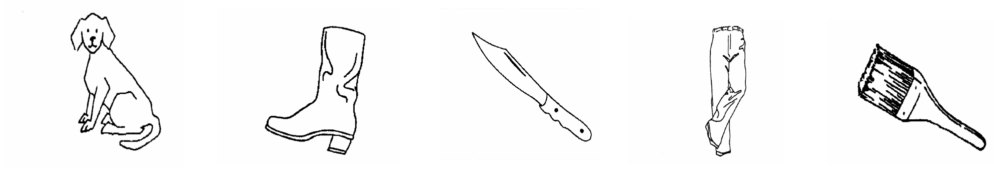

Step 2

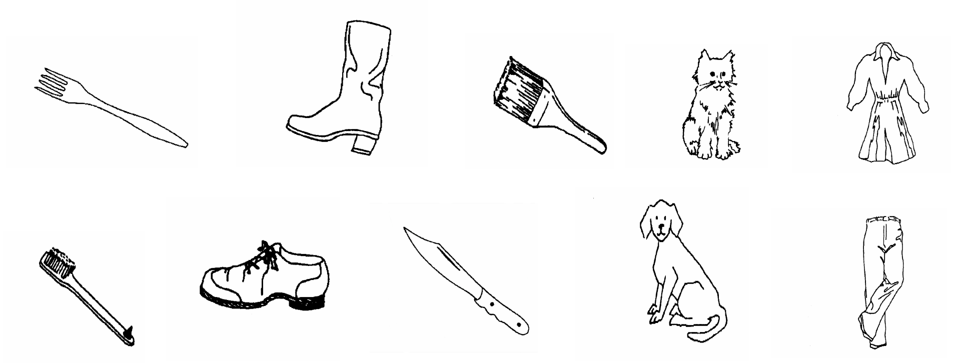

Visual ASE - Packet B

Step 1

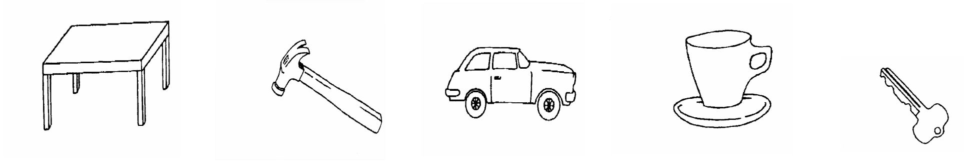

Step 2

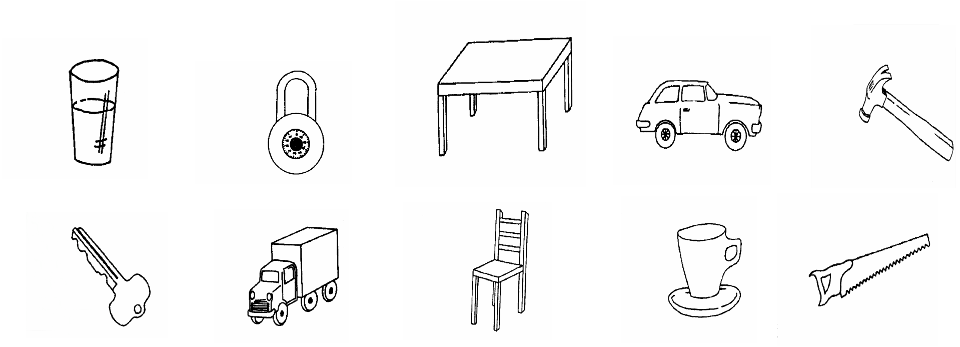

Supplement: Additional file 1: — CAM-ICU worksheet. The CAM-ICU worksheet is a concise description on how to perform the CAM-ICU in a standardized manner. [file 13054_2014_477_MOESM1_ESM.pdf]
